# Noise-induced Outer Hair Cells' Dysfunction and Cochlear Damage in Rabbits

**Published:** 2012-10-30

**Authors:** S A Moussavi-Najarkola, A Khavanin, R Mirzaei, M Salehnia, A Muhammadnejad, M Akbari

**Affiliations:** 1 Dept. of Occupational Health, School of Medical Sciences, Tarbiat Modares University (TMU), Tehran, Iran; 2 Dept. of Occupational Health, Health promotion research center, Zahedan University of Medical Sciences (ZUMS), Zahedan, Iran; 3 Dept. of Anatomical Sciences, School of Medical Sciences, Tarbiat Modares University (TMU), Tehran, Iran; 4 Cancer Research Center, Iran Cancer Institute, Tehran University of Medical Sciences (TUMS), Tehran, Iran; 5 Dept. of Audiology, School of Rehabilitation, Iran University of Medical Sciences (IUMS), Tehran, Iran

**Keywords:** Noise-induced Hearing Loss, Outer hair cells' function, Cochlear damage, Distortion product Otoacoustic emissions

## Abstract

**Background:**

Outer hair cells' (OHCs') dysfunctions as the extent of temporary and permanent threshold shifts (TTS and PTS) and cochlear damage were assessed in rabbits exposed to continuous noise

**Methods:**

Twelve New Zealand white rabbits were studied in noise (N) (n=6; exposed to continuous noise; 95 dB SPL, 500-8000 Hz for 8 h per day during 5 consecutive days) and control (C) (n=6; not exposed to noise). OHCs' functions were assessed by distortion product otoacoustic emission (DPOAE) level (Ldp) measurements in different periods and comparing TTS and PTS. Animals were anaesthetized by CO2; cochleae were extracted, fixed in 10% formaldehyde for 48 hours, decalcified by 10% nitric acid for 24 hours, and dehydrated, embedded, sectioned 5 µm thickness and stained by Hematoxylin and Eosin for light microscopy.

**Results:**

The most and least Ldp or TTS or PTS were related to 5888.50 Hz and 588.00 Hz respectively in noise subjected rabbits (P<0.05). TTS and PTS were decreased up to 17.79 dB and to 16.01 dB respectively. TTS were more than PTS over all test frequencies, especially at 5888.50 Hz (P<0.05). Ldp or TTS or PTS were found to be equal across ears (P>0.05). Severely vacuolated OHCs, pyknotic IHCs, swollen SC, and slightly thickened BM were found.

**Conclusion:**

Continuous noise extensively led to OHCs' dysfunctions as decreased Ldp (both TTS and PTS) and highly damage to cochlea.

## Introduction

Noise-induced hearing loss (NIHL) is referred as the most common potentially preventable form of sensorineural hearing impairment in industries (1). Most of conducted studies regarding NIHL are mainly related to continuous noise exposure (1). It must also be emphasized that noise exposures in life, environment, and industries are mostly as continuous noise exposure (2). Continuous noise exposure can cause temporary or permanent damage to the auditory system (3). So the ears have considerable comeback power from brief exposure to intense continuous noise and ordinarily recover within 24 hours to 48 hours, called as temporary threshold shift (TTS) (3). It must be considered that repeated or prolonged exposure to intense continuous noise gradually damages the cochlear hair cells of the inner ear, resulting in a permanent threshold shift (PTS) across multiple frequencies (4,5,6). Continuous noise exposure is believed that can induce higher TTS and PTS than intermittent noise exposure in animals and humans (7). Continuous noise over-stimulation can damage to the cochlea, hair cell membranes, and changes in size and shape of hair cells through different processes (6,7). Other effects of noise indicates include interference with communication, altered performance, annoyance, distraction, and interference with work or relaxation and physiological responses such as elevated blood pressure and sleep disturbances (8).

Whether or not continuous noise would alter hearing function or damage OHCs can be investigated on different laboratory animals (9). In order to assess the alterations and damage, distortion product otoacoustic emissions (DPOAEs) are assigned as a useful clinical tool for the early and differential diagnosis of damage to the OHCs in animals and humans (10-12). DP frequency is precisely related to the stimulus frequencies f_1_ and f_2_ by the formulas f_1_−N(f_2_−f_1_) for the lower band and f_2_+N(f_2_−f_1_) for the upper side band (13-16). In normal hearing, DPOAE-grams are close to each other at high and more separated at low stimulus levels, reflecting cochlear nonlinear sound processing (17-19). In cochlear hearing loss, DPOAE-grams are more separated even at high stimulus levels, revealing loss of cochlear amplifier compression (20). There are some limitations of L_dp_ recordings. First, electric microphone noise, physiological noise (breathing, blood flow) and external acoustic noise do not allow L_dp_ measurements at very low stimulus levels (20). Especially below 0.5 KHz, reliable L_dp_ measurements are not possible even at high stimulus levels (21-23). Second, because of the limited frequency range of the sound probe’s electroacoustic transducers, high-frequency L_dp_ measurements are difficult without using specialized devices (21,22). Third, standing waves in the outer ear canal make a defined stimulus setting difficult to obtain. Fourth, besides the main DPOAE source at f_2_, a secondary DPOAE source is present at the 2f_1_−f_2_ place, which interacts with the main source constructively or destructively at the f_2_ place (19,20). Therefore, DPOAE does not exactly reflect OHCs function at f_2_ place. There are also several technical aspects that must be considered in correct and acceptable DPOAE-gram recording (21,22,23). The most commonly used calibration method is the in-the-ear calibration based on the measurement of the sound-pressure level at the ear probe microphone for constant voltage at the loudspeaker (21,22). To access to maximum interaction site and preserve optimum overlap of the primary-tone traveling waves, the primary-tone level difference has to be increased with decreasing stimulus level, resulting in a L1│L2 setting described by L1=0.4L2+39.(22,23) The recording of L_dp_ requires the use of a highly sensitive low-noise microphone; loudspeakers need to exhibit a low distortion factor to minimize technical distortion; a tight fit of the probe is essential for L_dp_ recording; and the ear canal has to be clean and that the ear probe ports has not to be blocked with cerumen (21-23).

For better finding of outer hair cells' dysfunctions and cochlear damage caused by continuous noise and due to limitations in human studies, the present research was conducted to assessment outer hair cells' dysfunctions as the extent of temporary and permanent threshold shifts (TTS and PTS) and cochlear damage in rabbits exposed to continuous noise simulated to industrial situations.

## Materials and Methods

**Figure 1:**
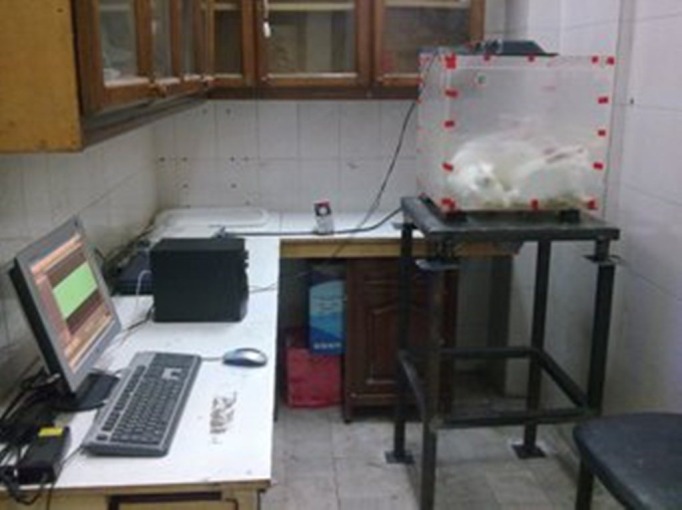
A cross-sectional view of exposure chamber (50×50×50 cm polycarbonate Plexiglas) has been shown. All rabbits were located into this chamber for exposure to noise pollutant (N group) and control group for 8 hr per day during 5 consecutive days. Exposure to noise has been carried out through a noise loudspeaker mounted on the roof of the chamber. Control group is just posed onto the chamber resembled to other groups with identical situations, but without any exposure

Twelve male New Zealand white (NZW) rabbits (2000±200 g body weight) were maintained in animal house at 20-22°C temperature, 30-70 % relative humidity, and 10 times/hour air displacement. Rabbits were fed to nutritional food and soft drink water. "General principles of Helsinki law related to laboratory animal" were used absolutely. Sample size was calculated 6 for any group according to pilot study. Noise group were exposed to 95 dBA SPL continuous noise at 500-8000 Hz for 8 hours per day during 5 consecutive days. Experimental protocol was such: baseline audiometry (day 0), rest periods (3 days; day 1 to 3), exposure periods (only for N group), secondary audiometry (an hour after latest exposure on day 8); rest period (3 days; day 9 to 11), and third audiometry (72 hours after latest exposure on day 11). Situations for control group were the same as noise group except for exposing to noise. Noise exposure was occurred in a transparent poly carbonated Plexiglas chamber dimensioned 50×50×50 cm based on calculating clearances needed for 6 rabbits, ventilated air volume, and reverberation environment (that SPL was independent on distances) (Figure 1).

Noise was delivered to animals in chamber equipped by a pair of loud speakers hanging on its roof. Noise was generated by means of Signal software manufactured by Pardisan Technology and Science Park, and delivered using Cool Edit Pro v. 2.1 manufactured by Syntrillium Software Corporation. Generated noise was amplified by an amplifier model ES-2000s manufactured by ES Audio Industrial Corporation, and propagated by a pair of loudspeakers type Micro Lab, model Subwoofer M-563 manufactured by Probit Company. SPL in chamber systematically monitored by Sound Level Meter (SLM type Precision) model CEL-490 manufactured by Cassella-CEL Company equipped to an analyzer located at animal hearing zone. Background noise in animal house and lab was below 20±2 dB.

Animals were anaesthetized by 60% Ketamine (40 mg/kg) and 40% Xylazine (10 mg/kg) mixture and examined otologically to exclude any infection or ear channel blocking wax. Middle ear health was examined by tympanometry. L_dp_ recording were done in left ear using DPOAE analyzer (DPOAE Model 4000 I/O, HOMOTH Company). L_dp_ audiograms were measured using two pure tone stimuli: f_1_–f_2_ with f_2_/f_1_ ratio of 1.25. Intensity levels of the two tones, L_1_ and L_2_ were equal to 75 and 65 dB SPL respectively. Before any L_dp_ recording, signal levels were calibrated in ear canal by using emission probe microphone. All data were collected into two stimuli; f_1_ and f_2_. Contents of these stimuli were summed, and summed energy in 2f_1_–f_2_ frequency buffer was served to estimate L_dp_ measurements at 0.5-10 KHz. Both L_dp_ and signal to noise ratio (SNR) were measured at 2f_1_–f_2_ and blotted respect to geometric mean of f_1_ and f_2_. Pass criterion for a valid signal evaluation procedure was typically set to SNR of 6 dB. Animals' body temperature was tried to keep constant during tests, since constant body temperature plays main role in L_dp_ measurement.

Animals were anaesthetized by carbon dioxide (CO_2_), decapitated, and their cochleae were extracted. Cochleae were fixed in 10% formaldehyde for 48 hours, decalcified by 10% nitric acid for 24 hours, dehydrated and cleared by Xylol. Specimens were embedded by paraffin in two-step; paraffin blocks were prepared and sectioned by 5 µm thickness by a calibrated precision microtome (Model Leitz). Sections were stained by Hematoxylin and Eosin (H&E). Cover slips were mounted on slides, left to dry and examined by light microscope (LM) (Zeiss model). Various segments of organ of corti in control group were histomorphologically examined under LM. Main parts involved in examination were inner hair cells (IHCs), outer hair cells (OHCs), supporting cells (SC), stria vascularis (SV), basilar membrane (BM), and tectorial membrane (TM). Noticeable parameters were cell size, relative cell count, inter- or intra-cellular distances, and cell polarity degree for each mentioned parts. It was allocated a score 0 to any parameter. Thus, control group was attributed as criteria for comparison. In the blind state, noise group were examined under LM at a magnification of 10×, 20× and 40×. Thus, any histomorphological damages of any parameter classified by scores -2, -1, 0, +1, and +2. Atrophy, edema, proliferation, and damages caused by cell injury were discriminated. Kolmogorov-Smirnov was used to determine data normality. Repeated Measures Analysis of Variance was served for comparing L_dp_ and L_nf_ among days 0, 8, and 11. One-way Analysis of Variance (ANOVA) was applied to multiple comparisons of L_dp_ and its L_nf_ at different frequencies. Tukey's Honestly Significant Difference as a Post hoc multiple comparisons were either used to determine differential L_dp_ and its L_nf_. Paired-Sample T-test was used to compare L_dp_ and its L_nf_ between right and left ears. Significant level was considered 0.05 as judgment.

## Results 

The pre- and post-exposure DPOAE levels (L_dp_) analysis showed that L_dp_ were found to be the same across days in control rabbits (P=0.065) (Table 1). L_dp_ were also equal over all test frequencies on each day (P=0.071). L_dp_ were showed to be the same between the right and left ears (P=0.068) (Table 1).

**Table 1:** Comparison of mean and standard deviation of DPOAE levels (L_dp_) and noise floor levels (L_nf_) across times in control group

Frequency (Hz)	DPOAE levels (L_dp_) (dB)					Noise floor levels (L_nf_) (dB)				
	Day 0	Day 8	Day 11		p	Day 0	Day 8	Day 11	p	
588.00	5.64 (0.12)	5.39 (0.15)	5.36 (0.18)	0.084		-0.97 (0.03)	-0.47 (0.05)	-0.88 (0.07)	0.062	
867.00	9.28 (0.11)	9.53 (0.09)	9.06 (0.15)	0.091		-1.21 (0.03)	-0.76 (0.07)	-1.48 (0.06)	0.059	
1133.00	13.12 (0.08)	13.34 (0.17)	13.40 (0.11)	0.318		-1.53 (0.05)	-2.75 (0.02)	-2.11 (0.03)	0.074	
1677.00	18.56 (0.28)	18.29 (0.21)	18.80 (0.17)	0.090		-3.17 (0.09)	-3.42 (0.11)	-2.03 (0.06)	0.053	
1967.00	23.21 (0.19)	23.45 (0.22)	23.25 (0.31)	0.067		-2.21 (0.04)	-3.24 (0.9)	-2.58 (0.07)	0.081	
3098.50	27.28 (0.42)	27.55 (0.37)	27.42 (0.45)	0.088		-3.45 (0.04)	-3.28 (0.08)	-4.87 (0.10)	0.411	
3956.00	31.77 (0.31)	31.49 (0.42)	31.99 (0.34)	0.129		-3.02 (0.09)	-3.13 (0.07)	-4.16 (0.16)	0.129	
5888.50	36.11 (0.43)	36.26 (0.32)	36.38 (0.37)	0.058		-4.91 (0.014)	-4.02 (0.11)	-4.79 (0.14)	0.056	
8166.50	34.89 (0.32)	34.98 (0.55)	34.75 (0.43)	0.066		-4.83 (0.10)	-4.26 (0.15)	-5.52 (0.18)	0.081	
9855.00	33.99 (0.42)	33.73 (0.57)	33.84 (0.53)	0.062		-5.74 (0.14)	-4.09 (0.13)	-5.36 (0.08)	0.059	

The most and least post-exposure L_dp_ were related to 5888.50 Hz and 588 Hz respectively in noise rabbits (Table 2). L_dp_ were decreased on days 8 and 11, significantly on day 8, in rabbits exposed to noise compared to control rabbits (P=0.006). Decreased L_dp_ at 5888.50 Hz were found to be more than other test frequencies (P<0.001). L_dp_ were found to be the same across ears (P=0.071). (Table 2)

**Table 2:** Comparison of mean and standard deviation of DPOAE levels (L_dp_) and noise floor levels (L_nf_) across times in noise group

Frequency (Hz)	DPOAE levels (L_dp_) (dB)					Noise floor levels (L_nf_) (dB)				
	Day 0	Day 8	Day 11		p	Day 0	Day 8	Day 11	p	
588.00	5.16 (0.08)	0.58 (0.02)	1.95 (0.10)	0.013		-5.11 (0.04)	-6.15 (0.08)	-6.73 (0.07)	0.091	
867.00	8.87 (0.12)	3.24 (0.26)	3.68 (0.23)	0.001		-6.68 (0.08)	-6.19 (0.10)	-6.06 (0.06)	0.077	
1133.00	13.08 (0.15)	6.39 (0.27)	6.85 (0.21)	0.008		-7.23 (0.06)	-6.63 (0.12)	-6.97 (0.05)	0.179	
1677.00	18.65 (0.32)	11.27 (0.22)	11.91 (0.35)	0.022		-7.04 (0.15)	-6.49 (0.11)	-7.75 (0.13)	0.088	
1967.00	23.14 (0.26)	12.82 (0.38)	14.63 (0.45)	0.016		-7.36 (0.17)	-8.96 (0.15)	-6.28 (0.17)	0.452	
3098.50	27.82 (0.38)	15.72 (0.43)	16.79 (0.29)	0.031		-8.32 (0.13)	-7.38 (0.16)	-8.56 (0.12)	0.089	
3956.00	31.18 (0.44)	18.01 (0.50)	19.10 (0.31)	0.002		-9.44 (0.12)	-8.21 (0.15)	-9.19 (0.16)	0.057	
5888.50	36.87 (0.53)	19.08 (0.41)	20.86 (0.35)	0.011		-9.23 (0.17)	-9.55 (0.13)	-8.88 (0.19)	0.266	
8166.50	34.96 (0.47)	17.74 (0.27)	19.28 (0.33)	0.009		-11.62 (0.18)	-10.77 (0.16)	-10.09 (0.17)	0.085	
9855.00	33.25 (0.39)	17.04 (0.49)	18.45 (0.41)	0.010		-11.04 (0.13)	-11.11 (0.15)	-12.71 (0.19)	0.151	

The most and least temporary threshold shifts (TTS) or permanent threshold shifts (PTS) were related to 5888.50 Hz and 588.00 Hz respectively in noise exposed rabbits (p=0.005) (Table 3). TTS and PTS were decreased up to 17.79 dB and to 16.01 dB respectively. TTS were more than PTS over all test frequencies, especially at 5888.50 Hz in noise rabbits (P=0.015). TTS or PTS in rabbits subjected to noise were larger than those in control rabbits (P<0.05). TTS or PTS were found to be equal across ears in noise exposed rabbits (P=0.071).

**Table 3:** Comparison of temporary threshold shifts (TTS) and permanent threshold shifts (PTS) between noise and control groups

Frequency (Hz)	Temporary threshold shifts (TTS) (dB)			Permanent threshold shifts (PTS) (dB)		
	Control group	Noise group	p	Control group	Noise group	p
588.00	0.25 (0.03)	4.58 (0.06)	0.032	0.28 (0.02)	3.21 (0.09)	0.002
867.00	0.25 (0.05)	5.63 (0.08)	0.021	0.22 (0.02)	5.19 (0.10)	0.005
1133.00	0.22 (0.04)	6.69 (0.11)	0.017	0.28 (0.01)	6.23 (0.09)	0.023
1677.00	0.27 (0.01)	7.38 (0.13)	0.011	0.24 (0.02)	6.74 (0.16)	0.019
1967.00	0.24 (0.05)	10.32 (0.14)	0.007	0.04 (0.01)	8.51 (0.12)	0.033
3098.50	0.27 (0.02)	12.10 (0.17)	0.003	0.14 (0.01)	11.03 (0.21)	0.020
3956.00	0.28 (0.02)	13.17 (0.12)	0.029	0.22 (0.04)	12.08 (0.19)	0.017
5888.50	0.15 (0.01)	17.79 (0.19)	0.003	0.27 (0.02)	16.01 (0.22)	0.018
8166.50	0.09 (0.05)	17.22 (0.13)	0.006	0.14 (0.02)	15.68 (0.17)	0.025
9855.00	0.26 (0.02)	16.21 (0.16)	0.014	0.15 (0.01)	14.80 (0.18)	0.019

**Figure 2:**
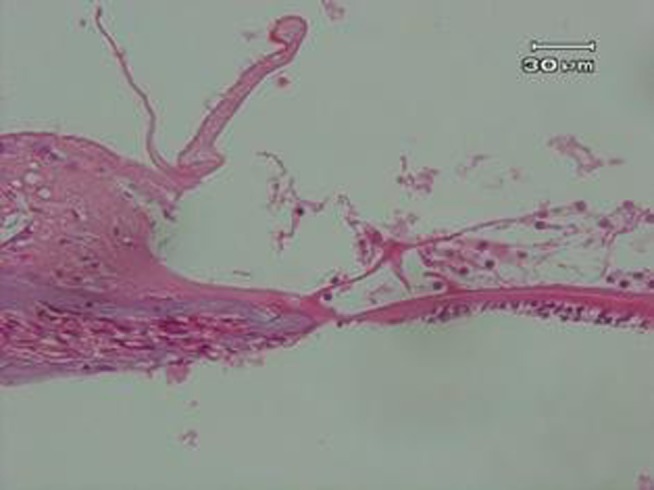
Control group. A photograph of the organ of corti of rabbits not exposed to any physical agents, showing healthy and normal cochlear hair cells (OHCs and IHCs), supporting cells (SC), basilar membrane (BM), and tectorial membrane (TM)

**Figure 3:**
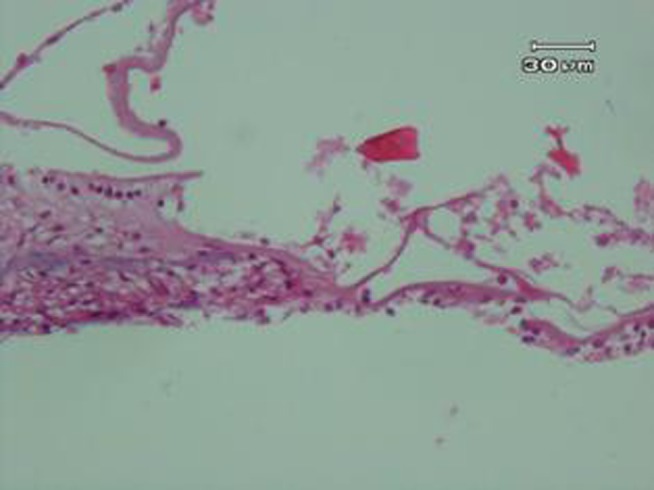
Noise group. A photograph of the organ of corti of rabbits exposed to noise, showing severely vacuolated outer hair cells (OHCs) with intensely cell injury as hydropic degeneration type, mild to moderately pyknotic inner hair cells (IHCs), swollen supportive cells, slightly thickened basilar membrane (BM), and not injured tectorial membrane (TM)

Control group examination showed normal cochlea (Figure 2). Therefore, there were no abnormal cases in examination of all slides of this group under microscopic observation. While severely vacuolated OHCs as well as intensively cell injury as hydropic degeneration type was obvious in noise group (Figure 3). Mild to moderately pyknotic inner hair cells (IHCs) were varied in some slides, but this state was not confirmed in all slides. SC was swollen, but not vacuolated. No status is found to be implying to injured and damaged TM, but slightly thickened has been shown.

## Discussion

Significantly decreased DPOAE levels (L_dp_) caused by noise exposure reached up to 20.86 dB (for day 8) and 19.08 dB (for day 11) at 5888.50 Hz. Like this study, most studies were indicated that prolonged and repeated exposure of awake animals to continuous noise led to significantly diminished L_dp_ at a wide test frequencies range as a reduction in cochlear outer hair cells' function depending on exposure duration, frequency and noise intensity (13-18,24,25). The difference in affected frequencies in the present study with other similar researches can be referred to the use of broad-band noise, while most of the others used narrow-band or pure tone stimulation in their efforts (24,25).

Contrary to these findings, several factors were found to be culprit in inducing enhanced DPOAE response amplitudes such as hypoxia, low frequency electromagnetic fields, induced labyrinthitis, and some ototoxic drugs (11,26). Consistent with the findings of the study, some studies showed that the DPOAE response amplitudes were significantly depressed following a number of factors include the administration of ototoxic drugs, acoustic trauma or noise overexposure, Meniere’s disease, sudden idiopathic sensori-neural hearing loss, acoustic neuroma, presbycusis, and hereditary hearing disorders (26,27).

DPOAE levels (L_dp_) were found to be significantly different on various occasions. L_dp_ decreased on day 8, and then increased at a level slightly higher than baseline measurements on day 11. Similar reversible and temporary differences were reported after interrupting the exposure to different noxious agents such as noise overexposure or acoustic trauma, ototoxic drugs, sudden idiopathic sensori-neural hearing loss, and thermoprobe lesioning (11,26). These decreases in DPOAE levels (L_dp_) might be attributed to the temporary and reversible effect of the vibration exposure as a basal cochlear lesion progressed through the frequency region being monitored. Consistently, some confirm that the temporary reduction in DPOAE amplitudes occurring before enhancements can be interpreted as relating to an improvement of the general condition of the exposed rabbits over time (26,27).

TTS and PTS were significantly decreased up to 17.79 dB and 16.01 dB respectively in animals under exposure to continuous noise. Like the results obtained from this study, PTS may be caused by a brief exposure to extremely high-intensity sounds, but it is more commonly caused by prolonged repetitive exposure or continuous exposure to lower levels of hazardous noise (4-6,27). Susceptibility to NIHL is highly variable; while some individuals are able to tolerate high noise levels for prolonged periods of time, others who are subjected to the same environment more rapidly lose hearing (27). Risk of PTS is related to the duration and intensity of the exposure as well as to genetic susceptibility to noise trauma (4,27). Inner ear is believed that partially protected from the effects of continuous noise by the acoustic reflex which is triggered when the ear is subjected to noise louder than 90 dB, causes the middle ear muscles (the stapedius and tensor tympani) to contract and thereby stiffen the conductive system, making it more resistant to sound entry (4). Because this protective reflex is neurally mediated, it is delayed in onset for a period ranging from 25 ms to 150 ms, depending on noise intensity (4).

Very highly vacuolation and intensively cell injury with the type of hydropic degeneration in outer hair cells (OHCs), mild to moderately pyknotic inner hair cells (IHCs), swollen supportive cells (SC), slightly thickened basilar membrane (BM) were found in noise group. Reasons for reduced L_dp_ is believed that can be attribute to misalignment of hair bundles on adjacent hair cells, non-linearity in stiffness of stereocilia, and damage of the tectorial membrane (2-4,9,28,29). Most studies found that the noise exposure causes permanent loss of hair cell stereocilia with apparent fracture of the rootlet structures and destruction of the sensory cells, which are replaced by nonfunctioning scar tissue. NIHL results from trauma to the sensory epithelium of the cochlea (4,9,28). In TTS, several potentially reversible effects such as regional decrease in stiffness of stereocilia secondary to contraction of rootlet structures which are anchored to the cuticular plate of hair cells, intracellular changes within the hair cells including metabolic exhaustion and microvascular changes, edema of the auditory nerve endings, and degeneration of synapses within the cochlear nucleus, can be occurred (2-4,9,28). While in PTS, the changes become irreversible and include breaks in the rootlet structures, disruption of the cochlear duct and organ of corti causing mixing of endolymph and perilymph, loss of hair cells, and degeneration of cochlear nerve fibers (2-4).

A strongly reason for cochlear OHCs' dysfunction (as decreased L_dp_) and damage to organ of corti is based on oxidative stress mechanism (30-33), Metabolic damage or exhaustion is believed that occurred when toxic waste products so-called as free radicals (FR_s_), including reactive oxygen species (ROS) or reactive nitrogen species (RNS), are formed after cochlear cells are stressed by reductions in cochlear blood flow, excessive and toxic levels of neurotransmitters like glutamate, changes in calcium balances in the cell, and other stress-related changes that are induced by noise (30-33). These free radicals injure a wide variety of critical structures in the cochlea, causing cell damage and cell death (32,33). Noise exposure affects several structural elements in hair cells, including the cell membrane and intracellular biochemical pathways (28). These changes may evoke the formation of free radicals, resulting in sensorineural hearing loss (33-37). FR_s_ may increase dramatically within a few minutes or hours of an intense noise exposure (30,38,39). Noise-induced cochlear FR_s_ endanger HC’s intrinsic antioxidant system as GSH that is found to be the powerful natural antioxidant glutathione peroxidase system in cochlear hair cells. Depletion of cochlear hair cells' GSH in organ of Corti due to exposure to noise can cause more susceptibility to hearing loss (38,39).

No any significance was observed about DPOAEs levels (L_dp_) between right and left ear in animals exposed to noise. Creation of reverberation field in exposure chamber seems to be the most important reason. Some studies have been reported results similar (9,25,28), but some reported different results regarding L_dp_ between two ears (40-42). Sato et al. (1991) showed that an efferent influence may also help to explain the systematic difference between the magnitude of left and right ear L_dp_ in humans and animals (40). Sininger & Cone-Wesson (2004) also indicated that tone-evoked L_dp_ are larger in left ear (41). van den Brink, (1970) reported pitch differences between left and right ears when presented with the same frequency stimulus (42).

L_dp_ measurements were examined in New Zealand white (NZW) rabbits as a species of rabbits experimented in this study, while the role of species differences must be taken as an important factor. It has been proved that there are clear species differences in the dependence of L_dp_ on frequency, in that L_dp_ tend to be largest in the regions of best hearing sensitivity in each species, and these regions vary between species (43). It has been reported that systematic variations in DPOAEs parameters such as L_1_=L_2_ and L_1_-L_2_, and f_2_/f_1_ generally produce qualitatively similar changes in emission levels in humans, monkeys, cats, rabbits, and rodents (23). They believes that these similarities occur despite the quantitative differences in particularly the f_2_/f_1_ ratio that elicits the largest DPOAEs, which is greater in rabbits and rodents (1.25) than in humans (1.22) (23).

Sex differences seem to play a key role in measuring L_dp_, while only male rabbits were used in present study. Some reported L_dp_ are larger in human and rhesus monkey females than in males (40,44). They found that the larger L_dp_ may be correlated to better hearing thresholds for females of the same species (43,44). Some are believed that this difference partly referred to different hormonal exposure (40,43), while others thought it can be attributed to a sex difference in OHC electromotility and/or in the mechanism(s) responsible for stereociliary bundle motility (40,44). Both of these reasons can be the result of gender differences in membrane lipid profiles that would alter lipid–protein interactions (44). A research cited that another possibility is the shorter length of female cochleae (40), or gender differences in the size of the middle ear (40,44). L_dp_ is expected to be varied or larger if the studied animals were selected females or variety of both male and female rabbits. A study reported L_dp_ is slightly stronger in female animals as compared to males (44).

DPOAEs can be attributed as a useful screening and diagnostic clinical tool for early detecting NIHL in rabbits with normal audiograms. Outer hair cells were affected early in NIHL, and DPOAEs were detected subtle changes in OHCs' function as temporary or permanent hearing shifts and cochlear damage. L_dp_ temporarily and permanently diminished in rabbits that underwent exposure to noise. Therefore, DPOAEs are an attractive tool for obtaining information about small temporary or permanent threshold shifts, even when the pure tone audiogram is normal. Noise exposure led to decreased L_dp_ and injury to IHCs, OHCs, SC, and BM. These cochlear dysfunction and histological changes seem to be the main reason for explaining the noise-induced hearing loss in rabbits subjected to excessive continuous noise.
